# When More Is Not Always Better: Linear and Nonlinear Predictors of Maternal Wellbeing During Pregnancy

**DOI:** 10.3390/bs16081380

**Published:** 2026-08-11

**Authors:** Risvan Vlad Rusu, Dan Octavian Rusu, Cristian Delcea

**Affiliations:** 1 Department of Research, Institute of Sexology, 400394 Cluj-Napoca, Romania; risvanvladrusu@sexology.ro; 2 Department of Applied Psychology, Babes-Bolyai University of Cluj-Napoca, 4305849 Cluj-Napoca, Romania; dan.rusu@ubbcluj.ro; 3 Multidisciplinary Doctoral School, Vasile Goldiș Western University of Arad, 310025 Arad, Romania

**Keywords:** maternal wellbeing, pregnancy, self-control, social support, perceived stress, ADHD symptoms, nonlinear effects, Too-Much-of-a-Good-Thing, maternal mental health, second trimester

## Abstract

Maternal wellbeing during pregnancy is an important component of maternal mental health and has implications for both maternal and child outcomes. Although psychological resources such as self-control and social support are generally assumed to promote wellbeing, maternal wellbeing may also be influenced by psychological vulnerabilities, including perceived stress and ADHD (Attention Deficit Hyperactivity Disorder) symptoms. Recent theoretical perspectives further suggest that some beneficial characteristics may exhibit diminishing returns at higher levels. This study examined linear and nonlinear associations among perceived stress, ADHD symptoms, self-control, perceived social support, and maternal wellbeing during the second trimester of pregnancy. A sample of 149 first-time pregnant women from Romania completed measures of maternal wellbeing, perceived stress, ADHD symptoms, self-control, and perceived support from partners, family members, and friends. Hierarchical regression analyses were conducted, including quadratic terms derived from the Too-Much-of-a-Good-Thing framework. Perceived stress emerged as the strongest predictor of lower maternal wellbeing, whereas family support was positively associated with wellbeing. ADHD symptoms were negatively associated with wellbeing at the bivariate level but did not uniquely predict wellbeing after controlling for the remaining study variables. Most importantly, self-control demonstrated a significant nonlinear association characterized by diminishing returns, such that increases in self-control were associated with progressively smaller improvements in wellbeing at higher levels. No comparable nonlinear effects were observed for perceived stress or social support. Overall, the findings suggest that maternal wellbeing is shaped by both psychological vulnerabilities and psychological resources, and that the benefits of some psychological strengths may not increase uniformly across their entire range.

## 1. Introduction

### 1.1. Maternal Wellbeing During the Second Trimester of Pregnancy

Maternal mental health during pregnancy is increasingly recognized as a major public health concern because of its implications for maternal functioning, obstetric outcomes, infant development, and early parent–child relationships (Glover, 2014; Howard & Khalifeh, 2020; Howard et al., 2014). Importantly, maternal mental health extends beyond the absence of psychopathology and includes positive psychological functioning, emotional balance, vitality, and the ability to adapt effectively to pregnancy-related changes (World Health Organization [WHO], 2022). This perspective is consistent with broader models of positive mental health, which conceptualize wellbeing as a distinct construct rather than merely the absence of distress (Diener et al., 1999; Keyes, 2002; Ryff, 1989). Understanding maternal wellbeing is therefore important because women may experience reduced vitality, impaired coping, or diminished psychological adjustment even in the absence of clinically significant depression or anxiety.

The second trimester represents a particularly relevant period for studying maternal wellbeing. Compared with the first trimester, which is often characterized by physical discomfort, uncertainty, and concerns about pregnancy viability, the second trimester is generally associated with greater physiological stability and psychological adjustment (Dunkel Schetter, 2011; Guardino & Dunkel Schetter, 2014). During this period, fetal movement is commonly first perceived, the pregnancy becomes increasingly visible, and maternal–fetal attachment typically strengthens, contributing to a more consolidated maternal identity and greater psychological engagement with the pregnancy (Alhusen, 2008; Cannella, 2005; Guo & Dipietro, 2010; Slade et al., 2009). At the same time, women often experience increased involvement from partners, family members, and friends, making this stage particularly suitable for examining how psychological vulnerabilities and psychosocial resources are associated with wellbeing. Furthermore, mid-pregnancy has been identified as a potentially important window for preventive interventions, as psychological resources and coping processes become increasingly relevant before the demands of late pregnancy and childbirth emerge (Dunkel Schetter, 2011; Lobel et al., 2008; Woods et al., 2010; Valdebenito et al., 2020; Van de Glind et al., 2013; Yerkes & Dodson, 1908).

Restricting the study to the second trimester was an intentional design decision. Although pregnancy symptoms vary across gestation, the first trimester is characterized by substantial physiological and obstetric instability, including nausea and vomiting that typically begin between 6 and 8 weeks of gestation and the highest concentration of pregnancy losses (Genovese & McQueen, 2023; Jarvis & Nelson-Piercy, 2011). In contrast, the third trimester is increasingly influenced by sleep disturbances, musculoskeletal discomfort, and childbirth-related anxiety, all of which may independently affect maternal wellbeing (Dennis et al., 2017; Ji et al., 2024; Mislu et al., 2024). Longitudinal evidence further indicates that pregnancy symptoms follow systematic trajectories across gestation, with early somatic symptoms predominating during the first trimester and sleep-related complaints becoming progressively more common toward the end of pregnancy (Nissen et al., 2023). Restricting the sample to the second trimester therefore reduced gestational-stage heterogeneity and allowed maternal wellbeing to be examined within a comparatively less confounded period of pregnancy. This design decision was also consistent with the objectives of the present study, as greater sample homogeneity may facilitate the estimation and interpretation of nonlinear associations, which are generally more difficult to detect than linear effects (A. H. Diallo et al., 2004; Grant & Schwartz, 2011).

Event Romanian evidence further underscores the clinical relevance of examining psychological wellbeing during pregnancy. In a sample of primiparous women assessed between 20 and 28 weeks of gestation, perceived stress, depression, anxiety, and resilience were associated with obstetric outcomes at the bivariate level, although generalized anxiety remained the only independent psychological predictor after mutual adjustment (Socol et al., 2025). In addition, a large Romanian study of pregnant women found that quality of life was associated with educational attainment, employment status, and partner support, highlighting the contribution of both socioeconomic and relational resources to maternal adjustment. Together, these findings support examining psychological vulnerabilities and psychosocial resources within an integrated framework and demonstrate the relevance of maternal wellbeing as a research and clinical concern in the Romanian context.

### 1.2. Psychological Vulnerabilities and Maternal Wellbeing

During pregnancy, psychological vulnerabilities such as perceived stress and ADHD symptoms may undermine adaptive functioning and maternal wellbeing by impairing women’s capacity to manage internal and external demands during a period of substantial change (Cohen et al., 1983; Faraone et al., 2021). Perceived stress is one of the most robust psychological predictors of poorer maternal mental health during pregnancy. Defined as the subjective appraisal of life circumstances as unpredictable, uncontrollable, or overwhelming (Cohen et al., 1983; Lazarus & Folkman, 1984), perceived stress has been consistently associated with antenatal depression, anxiety, emotional distress, and reduced wellbeing (Biaggi et al., 2016; Lancaster et al., 2010; Robertson et al., 2004). Beyond its psychological correlates, prenatal stress has also been linked to biological stress-regulation processes and adverse maternal and child outcomes (Glover, 2014; O’Donnell & Meaney, 2017; Van den Bergh et al., 2020). Accordingly, because higher perceived stress reflects greater difficulty coping with pregnancy-related demands, women reporting higher levels of perceived stress were expected to report lower levels of maternal wellbeing (Biaggi et al., 2016; Lancaster et al., 2010).

ADHD symptoms may represent an important psychological vulnerability during pregnancy because they can interfere with the self-regulatory demands associated with prenatal adaptation. Difficulties in attention, organization, planning, emotion regulation, and stress management may complicate engagement with prenatal care, processing of health-related information, and adjustment to the physical and psychological changes of pregnancy (Faraone et al., 2021; Kooij et al., 2019). Furthermore, women with ADHD are at increased risk for anxiety, depression, sleep problems, and other psychosocial difficulties that may adversely affect maternal wellbeing (Kooij et al., 2019; Shaw et al., 2014).

Emerging evidence suggests that ADHD is also associated with poorer perinatal mental health outcomes. Population-based studies have reported elevated risks of perinatal depression, anxiety, and postpartum affective disorders among women with ADHD (Andersson et al., 2023; Baker et al., 2022). Although research on ADHD during pregnancy remains relatively limited, mechanisms such as emotion dysregulation, executive-function difficulties, and increased stress sensitivity are likely relevant throughout the prenatal period. Accordingly, ADHD symptoms were expected to be negatively associated with maternal wellbeing (Dennis & Ross, 2006; Dennis & Letourneau, 2007; Dunkel Schetter & Tanner, 2012; Egan et al., 2011; S. C. Hayes et al., 2006).

### 1.3. Self-Control as a Psychological Resource

Although previous research has generally reported positive linear associations between self-control and psychological adjustment (Hofmann et al., 2014; Tangney et al., 2004; Wiese et al., 2018), several theoretical perspectives suggest that the benefits of self-control may not increase indefinitely. According to the Too-Much-of-a-Good-Thing (TMGT) framework, characteristics that are generally advantageous may become less beneficial or even counterproductive when expressed at very high levels (Pierce & Aguinis, 2013). Applied to self-control, this perspective proposes that excessive self-regulation may be associated with rigidity, excessive monitoring of thoughts and behaviors, reduced spontaneity, and diminished psychological flexibility (Grant & Schwartz, 2011). Wiese et al. (2018) further noted that very high self-control could theoretically involve frequent and unnecessary regulation of emotions, thoughts, and behaviors, potentially resulting in a more constrained and less rewarding psychological experience (Risser et al., 2024; Stein et al., 2014; Moffitt et al., 2011; O’Hara & McCabe, 2013).

These considerations may be particularly relevant during pregnancy, a developmental period characterized by uncertainty, rapidly changing circumstances, and the need for flexible adaptation. While moderate-to-high levels of self-control may facilitate coping with pregnancy-related demands, extremely high levels could theoretically be associated with overcontrol, perfectionistic tendencies, or excessive self-monitoring, potentially limiting the psychological benefits typically associated with self-regulation. Importantly, no studies to date have directly examined whether the association between self-control and maternal wellbeing is nonlinear during pregnancy. Therefore, in addition to the expected positive linear association, the present study explored the possibility of a curvilinear relationship between self-control and maternal wellbeing (Leahy-Warren et al., 2012; Lee, 2012; Limburg et al., 2017; Brock & Lawrence, 2009; Collins et al., 1993; Adler et al., 2006).

### 1.4. Social Support During Pregnancy

Social support is a multidimensional construct, and evidence suggests that support from different sources may play distinct roles during pregnancy. Partner support has been consistently associated with lower antenatal distress, reduced risk of depression and anxiety, and better maternal adjustment (Biaggi et al., 2016; Stapleton et al., 2012). Family support may provide practical assistance, emotional reassurance, informational guidance, and caregiving resources during the transition to parenthood and has been linked to better maternal mental health outcomes (Ginja et al., 2018; Stapleton et al., 2012). Friend support, in turn, is often based on voluntary affiliation, companionship, and shared experiences and has been associated with greater emotional wellbeing, social connectedness, and reduced feelings of isolation during pregnancy and the perinatal period (Ginja et al., 2018; Hetherington et al., 2018). Although evidence generally supports the protective role of social relationships, the effectiveness of support may depend not only on its availability but also on whether it is perceived as responsive to the recipient’s needs and preferences (Bolger & Amarel, 2007; Maisel & Gable, 2009). Given these potentially distinct contributions, the present study examined partner, family, and friend support as separate dimensions rather than combining them into a single global measure of social support (Cranley, 1981; Crnic & Low, 2002; Hobfoll, 1989; Block & Block, 1980; Brock & Lawrence, 2009).

### 1.5. Beyond “More Is Better”: The Too-Much-of-a-Good-Thing Hypothesis

Most research on maternal wellbeing assumes that psychological and social resources are beneficial in a largely linear manner, such that greater resources are associated with better adjustment and mental health outcomes. Consistent with this perspective, higher levels of social support and self-regulatory capacities have generally been linked to greater wellbeing, resilience, and adaptive functioning (Biaggi et al., 2016; Hofmann et al., 2014; Tangney et al., 2004; Wiese et al., 2018; Baumeister et al., 2007).

However, the Too-Much-of-a-Good-Thing (TMGT) framework suggests that characteristics that are generally beneficial may become less advantageous when present at very high levels (Grant & Schwartz, 2011; Pierce & Aguinis, 2013). This possibility may be particularly relevant during the second trimester of pregnancy, a period characterized by increasing psychological engagement with the pregnancy, preparation for parenthood, and heightened involvement from significant others (Alhusen, 2008; Dunkel Schetter, 2011; Slade et al., 2009). During this period, social support may be most beneficial when it is responsive to the mother’s needs rather than experienced as intrusive or autonomy-limiting (Bolger & Amarel, 2007; Maisel & Gable, 2009). Similarly, while self-control may facilitate coping with pregnancy-related demands, excessively high levels could theoretically be associated with rigidity, perfectionistic tendencies, or reduced psychological flexibility, potentially limiting the benefits of self-regulation (Grant & Schwartz, 2011; T. R. Lynch, 2018). Taken together, these considerations suggest that the associations between psychological resources and maternal wellbeing during the second trimester may not always be strictly linear and warrant examination of potential nonlinear effects (Field, 2018; Block & Block, 1980; American Psychiatric Association, 2022).

### 1.6. This Study

This study examined the linear and nonlinear associations between perceived stress, ADHD symptoms, self-control, social support, and maternal wellbeing during the second trimester of pregnancy. Maternal wellbeing was assessed using the WHO-5 Well-Being Index, a widely used measure of positive psychological functioning (Topp et al., 2015). Perceived stress and ADHD symptoms were conceptualized as psychological vulnerabilities and were expected to show negative linear associations with maternal wellbeing. In contrast, self-control and support from partners, family, and friends were conceptualized as psychological and social resources for which both linear and nonlinear associations were examined. By focusing specifically on the second trimester and integrating psychological vulnerabilities and resources within a common conceptual framework, this study extends previous research by examining whether the benefits of key psychological and social resources increase continuously or exhibit diminishing returns at higher levels, as suggested by the Too-Much-of-a-Good-Thing perspective.

In addition to these linear associations, nonlinear relationships were examined for perceived stress, self-control, and social support. Although most perinatal studies assume a linear detrimental effect of stress, research conducted in other developmental and community samples suggests that stress-related experiences may sometimes exhibit curvilinear associations with psychological functioning, with moderate levels associated with more favorable outcomes than either very low or very high levels (Arbel et al., 2020; Han et al., 2023; Seery et al., 2010). Likewise, the Too-Much-of-a-Good-Thing framework suggests that the benefits of psychological and social resources may diminish at higher levels rather than increasing indefinitely (Grant & Schwartz, 2011; Pierce & Aguinis, 2013). Accordingly, we examined whether nonlinear patterns characterized by diminishing returns would emerge for perceived stress, self-control, and social support during the second trimester of pregnancy.

Based on these considerations, the following hypotheses were formulated:

**H1.**  *Higher perceived stress will predict lower maternal wellbeing.*

**H2.**  *Higher ADHD symptoms will predict lower maternal wellbeing.*

**H3.**  *Self-control will exhibit a nonlinear positive association with maternal wellbeing such that increases in self-control will be associated with progressively smaller improvements in wellbeing at higher levels of self-control.*

**H4.**  *Partner support will exhibit a nonlinear positive association with maternal wellbeing such that increases in partner support will be associated with progressively smaller improvements in wellbeing at higher levels of partner support.*

**H5.**  *Family support will exhibit a nonlinear positive association with maternal wellbeing such that increases in family support will be associated with progressively smaller improvements in wellbeing at higher levels of family support.*

**H6.**  *Friend support will exhibit a nonlinear positive association with maternal wellbeing such that increases in friend support will be associated with progressively smaller improvements in wellbeing at higher levels of friend support.*

**H7.**  *Perceived stress will exhibit a nonlinear association with wellbeing, such that wellbeing may be relatively preserved or even slightly higher at low to moderate levels of perceived stress, but will decrease at higher levels.*

## 2. Materials and Methods

### 2.1. Participants

Participants were recruited between 2 April and 28 May 2026 as part of a broader study examining maternal wellbeing during pregnancy. No financial compensation was provided for participation.

A total of 173 women completed the survey. Twenty-four participants were excluded because they were not in the second trimester of pregnancy, resulting in a final sample of 149 women.

Eligibility criteria required participants to be at least 18 years old, currently pregnant with their first child, and in the second trimester of pregnancy at the time of survey completion. Although recruitment materials initially targeted women in early pregnancy, only participants who were in the second trimester when completing the questionnaire were retained for analysis. Exclusion criteria included a self-reported diagnosis of a severe psychiatric disorder (e.g., schizophrenia or bipolar disorder) and incomplete questionnaire responses.

### 2.2. Procedure

Participants were recruited online through a Facebook support group dedicated to women experiencing their first pregnancy. A study advertisement containing a brief description of the research aims and a link to the online questionnaire was posted within the group. Social media recruitment has become an increasingly common and cost-effective method for enrolling pregnant women in health and psychological research, allowing researchers to efficiently reach geographically diverse samples (Thornton et al., 2016).

Interested individuals accessed the questionnaire through a secure online survey platform. To reduce the likelihood of duplicate responses, the survey was configured to permit only one submission per device and IP address. Additionally, response patterns were reviewed for potential duplicates and inconsistencies, following recommendations for online survey research (Aust et al., 2013).

Before accessing the questionnaire, participants were presented with an electronic informed consent form describing the study objectives, confidentiality procedures, voluntary nature of participation, and their right to withdraw at any time without penalty. Only participants who provided informed consent were allowed to continue to the survey. No personally identifying information was collected, and all responses were recorded anonymously.

The study protocol was conducted in accordance with the ethical principles outlined in the Declaration of Helsinki (World Medical Association, 2013) and was reviewed and approved by the Ethics Committee of the Sexology Institute of Romania (Approval No. IS02549) on 10 March 2026.

### 2.3. Measures

Participants completed a single online survey comprising demographic questions and all study measures. The survey included standardized questionnaires assessing maternal wellbeing, perceived stress, ADHD symptoms, self-control, and perceived social support. All measures were administered in Romanian. Previously validated Romanian-language versions were used where available; adapted measures for which no formal Romanian validation was available are identified below.

To evaluate the internal consistency of the study measures, both Cronbach’s alpha (α) and McDonald’s omega (ω) were calculated. Cronbach’s alpha remains the most frequently reported index of internal consistency; however, it assumes essential tau-equivalence, meaning that all items contribute equally to the latent construct. Because this assumption is often violated in psychological instruments, alpha may underestimate or overestimate reliability depending on the underlying measurement model. In contrast, McDonald’s omega is based on a congeneric measurement model that allows items to contribute unequally to the latent construct and is therefore considered a more accurate estimate of composite reliability when item loadings differ. Consequently, both coefficients are reported to provide a comprehensive evaluation of scale reliability (A. F. Hayes & Coutts, 2020; Viladrich et al., 2017).

Following commonly accepted guidelines, reliability coefficients of 0.90 or higher were considered excellent, values between 0.80 and 0.89 good, between 0.70 and 0.79 acceptable, between 0.60 and 0.69 questionable but potentially adequate for exploratory research or brief scales, and values below 0.60 indicative of poor internal consistency requiring cautious interpretation (Nunnally & Bernstein, 1994; A. F. Hayes & Coutts, 2020).

**Maternal Wellbeing**

Maternal wellbeing was assessed using the World Health Organization-Five Well-Being Index (WHO-5; World Health Organization [WHO], 2022; Topp et al., 2015). The WHO-5 consists of five items assessing positive emotional wellbeing during the previous two weeks (e.g., “I have felt calm and relaxed”). Responses are rated on a six-point scale ranging from 0 (At no time) to 5 (All of the time), with higher scores indicating greater psychological wellbeing. Raw scores were multiplied by four to produce a total score ranging from 0 to 100. In a prospective study of 148 pregnant women, the WHO-5 demonstrated good internal consistency (Cronbach’s α = 0.81). Concurrent validity for antenatal depressive symptoms was supported by an area under the curve of 0.80 (95% CI [0.72, 0.87]), with sensitivity of 89.47% and specificity of 67.18%. WHO-5 scores also predicted postpartum depressive symptoms (AUC = 0.73, 95% CI [0.60, 0.86]; Do et al., 2021).

**Perceived Stress**

Perceived stress was measured using the 10-item Perceived Stress Scale (PSS-10; Cohen et al., 1983). The scale assesses the extent to which individuals perceive their lives as unpredictable, uncontrollable, and overwhelming during the previous month. Items are rated on a five-point scale ranging from 0 (Never) to 4 (Very often), with higher scores indicating greater perceived stress. In a prospective study of pregnant women, the PSS-10 demonstrated acceptable internal consistency (Cronbach’s α = 0.76) and discriminated antenatal depressive symptoms with an area under the curve of 0.84 (95% CI [0.76, 0.93]), sensitivity of 89.47%, and specificity of 65.65% (Do et al., 2021). Additional cross-cultural evidence was provided by Katus et al. (2022), who examined the PSS-10 in 1208 expectant mothers from eight countries, including Romania. Their analyses supported a two-factor structure and established configural and metric invariance across study sites, although scalar invariance across countries was not supported.

**ADHD Symptoms**

Maternal ADHD symptoms were assessed using the same five-item measure employed in the Evidence for Better Lives Study (EBLS) and described by Murray et al. (2022). The measure combines three items adapted from the Adult ADHD Self-Report Scale Screener (ASRS-v1.1) with two additional items adapted from the age-20 assessment of the Zurich Project on Social Development from Childhood to Adulthood (z-proso), selected to improve applicability to adult women. Participants responded on a five-point scale ranging from 1 (Never) to 5 (Always), with higher scores indicating greater ADHD symptomatology. In the EBLS cohort, this exact five-item composite demonstrated modest internal consistency (Cronbach’s α = 0.59; Murray et al., 2022). However, no published factor-analytic, convergent-validity, or diagnostic-accuracy statistics are available for this exact item configuration. For comparison, the original six-item ASRS-v1.1 screener demonstrated sensitivity of 68.7%, specificity of 99.5%, total classification accuracy of 97.9%, and agreement with clinical diagnosis of κ = 0.76 (Kessler et al., 2005). These estimates cannot be assumed to apply to the adapted five-item composite used in the present study, which was therefore treated as an ADHD symptom index rather than a diagnostic screening instrument.

**Self-Control**

Self-control was measured using the 13-item Brief Self-Control Scale (BSCS; Tangney et al., 2004). The BSCS assesses the dispositional ability to regulate thoughts, emotions, and behaviors and to resist short-term temptations in favor of longer-term goals (e.g., “I am good at resisting temptation”). Responses are provided on a five-point scale ranging from 1 (Not at all like me) to 5 (Very much like me), with higher scores indicating greater self-control. In two independent samples, the 13-item BSCS demonstrated internal consistency coefficients of α = 0.73 (95% CI [0.69, 0.78]) and α = 0.81 (95% CI [0.77, 0.85]), together with six-month test–retest reliability of r = 0.66. Convergent validity was supported by negative correlations with urgency (r = −0.42), lack of premeditation (r = −0.34), and lack of perseverance (r = −0.55; Brevers et al., 2017).

**Partner Support**

Perceived support from a romantic partner was assessed using a five-item measure of partner supportiveness adapted from the relationship-quality items used by Goldberg and Carlson (2014). Participants rated statements concerning the emotional and practical support provided by their partner on a five-point scale ranging from 1 (Strongly disagree) to 5 (Strongly agree), with higher scores reflecting greater perceived partner support. Published numerical estimates of reliability, factorial validity, or convergent validity were not identified for this exact five-item configuration, and the measure has not been formally validated in Romanian. In the present sample, the measure demonstrated acceptable internal consistency according to Cronbach’s alpha and good internal consistency according to McDonald’s omega (α = 0.75, ω = 0.84). Findings involving this adapted measure should therefore be interpreted cautiously pending further psychometric validation.

**Family and Friend Support**

Perceived support from family members and friends was assessed using the corresponding subscales of the Multidimensional Scale of Perceived Social Support (MSPSS; Zimet et al., 1988). The Family and Friends subscales each contain four items, which were rated in the present study on a five-point scale ranging from 1 (Strongly disagree) to 5 (Strongly agree). Higher scores indicate greater perceived support from the respective source. In the original validation study, the Family and Friends subscales demonstrated strong internal consistency (α = 0.87 and α = 0.85, respectively) and test–retest reliability over a two- to three-month interval (r = 0.85 and r = 0.75, respectively). Construct validity was supported by negative correlations between family support and depression (r = −0.24) and anxiety (r = −0.18), as well as between friend support and depression (r = −0.24; Zimet et al., 1988). A subsequent Romanian validation of the complete 12-item MSPSS supported its three-factor structure and reported high internal consistency for both the Family (α = 0.90) and Friends (α = 0.91) subscales (Alexe et al., 2021). Because the Romanian validation examined the complete MSPSS using its standard response format, its findings provide supportive rather than directly equivalent evidence for the modified five-point administration used in the present study.

Internal consistency estimates obtained for all measures in the present sample are reported in Section 3.2 and Table 1.

### 2.4. Data Analysis

Data analyses were conducted using IBM SPSS Statistics (Version 30). Prior to hypothesis testing, descriptive statistics, bivariate correlations, and internal consistency estimates were calculated for all study variables. Internal consistency was evaluated using both Cronbach’s alpha (α) and McDonald’s omega (ω). Although Cronbach’s alpha remains the most widely reported reliability coefficient, McDonald’s omega was also calculated because it provides a less restrictive estimate of internal consistency when the assumption of equal factor loadings is not met (Dunn et al., 2014; A. F. Hayes & Coutts, 2020).

Preliminary analyses were conducted to evaluate the assumptions underlying multiple regression. Distributions of all study variables were inspected using descriptive statistics, histograms, and normal probability plots. Multicollinearity was assessed using variance inflation factors (VIFs) and tolerance statistics. Standardized residuals, Cook’s distance values, Mahalanobis distances, and residual plots were examined to evaluate linearity, homoscedasticity, normality of residuals, and the presence of influential observations (Field, 2018; Hair et al., 2019; Tabachnick & Fidell, 2019).

To examine associations between the study variables and maternal wellbeing, hierarchical multiple regression analyses were performed. Maternal wellbeing (WHO-5) was entered as the dependent variable. In Model 1, age and maternal education were entered as control variables. In Model 2, perceived stress and ADHD symptoms were entered as psychological vulnerability factors. Self-control was added in Model 3, whereas perceived support from partners, family members, and friends was entered in Model 4. This sequence was selected to evaluate the incremental contribution of psychological resources and social support beyond demographic characteristics and psychological vulnerabilities.

To examine potential nonlinear associations, quadratic terms were subsequently entered in a final model. Consistent with the Too-Much-of-a-Good-Thing framework (Grant & Schwartz, 2011; Pierce & Aguinis, 2013), quadratic effects were tested for self-control and the three sources of social support (partner, family, and friend support). In addition, an exploratory quadratic term was estimated for perceived stress because previous research outside the perinatal literature has suggested that moderate levels of stress or adversity may sometimes be associated with more adaptive outcomes than either very low or very high levels of stress (Arbel et al., 2020; Seery et al., 2010).

Prior to computing quadratic terms, all continuous predictors involved in nonlinear analyses were mean-centered to reduce multicollinearity between linear and quadratic components (Aiken et al., 1991). Quadratic terms were then created by squaring the centered variables. Significant quadratic effects were interpreted as evidence of curvilinear associations and were further examined through graphical inspection of the fitted regression functions.

Given the fixed sample size, a sensitivity analysis was conducted using G*Power 3.1 within the fixed-model R^2^-increase framework (Faul et al., 2009). With *N* = 149, 13 predictors in the full model, α = 0.05, and 80% statistical power, the minimum detectable unique effect for an individual quadratic predictor was approximately *f*^2^ = 0.05, whereas the minimum detectable incremental effect for the five quadratic terms tested jointly was approximately *f*^2^ = 0.09. The study was therefore better powered to detect small-to-moderate nonlinear effects than very small quadratic associations.

To obtain more robust parameter estimates, bias-corrected and accelerated (BCa) bootstrap confidence intervals were calculated using 5000 bootstrap samples. Statistical significance was evaluated using a two-tailed alpha level of 0.05.

## 3. Results

### 3.1. Sample Characteristics

The final sample consisted of pregnant women in their second trimester of pregnancy ranging in age from 18 to 43 years (*M* = 29.99, *SD* = 4.63). Participants were recruited across several Romanian cities, with the largest proportion residing in Cluj-Napoca (39.6%), followed by Satu Mare (22.8%), Baia Mare (14.1%), Târgu Mureș (12.1%), and Bucharest (11.4%).

The sample included substantial ethnic diversity, with 58.4% of participants identifying as Romanian, 36.9% as Hungarian, and 4.7% as Roma. Educational attainment was generally high: 37.2% of participants held a bachelor’s degree, 33.8% held a master’s degree, and 2.7% reported doctoral-level education. Smaller proportions reported completing high school (13.5%), vocational or technical education (5.4%), secondary education (1.4%), or other forms of education (6.1%). Overall, the sample was ethnically diverse but relatively homogeneous in terms of high educational attainment.

### 3.2. Internal Consistency of Study Measures

Internal consistency estimates for all study measures are presented in Table 1. Because Cronbach’s alpha and McDonald’s omega rely on different measurement assumptions, both coefficients were calculated to provide a comprehensive evaluation of internal consistency. Whereas Cronbach’s alpha assumes essential tau-equivalence, McDonald’s omega provides a composite reliability estimate that is less sensitive to violations of this assumption and unequal item loadings (A. F. Hayes & Coutts, 2020; Viladrich et al., 2017).

Overall, the instruments demonstrated acceptable to excellent reliability. The WHO-5 Wellbeing Index showed good internal consistency (α = 0.84, ω = 0.84), as did the Perceived Stress Scale (PSS-10; α = 0.83, ω = 0.87). The partner support measure demonstrated acceptable to good reliability (α = 0.75, ω = 0.84).

The family support and friend support subscales of the Multidimensional Scale of Perceived Social Support (MSPSS) demonstrated excellent internal consistency (family support: α = 0.90, ω = 0.94; friend support: α = 0.93, ω = 0.95).

The ADHD symptom measure showed lower reliability according to Cronbach’s alpha but acceptable reliability according to McDonald’s omega (α = 0.56, ω = 0.74), likely reflecting the brevity and heterogeneous composition of the scale. Similarly, the self-control measure demonstrated modest reliability according to Cronbach’s alpha but acceptable reliability according to McDonald’s omega (α = 0.61, ω = 0.75). Taken together, these findings indicate that the majority of the study measures demonstrated acceptable to excellent internal consistency, with higher omega coefficients than alpha observed for the ADHD symptom and self-control measures.

### 3.3. Descriptive Statistics

Descriptive statistics and bivariate correlations among all study variables are presented in Table 2. Participants reported relatively high levels of maternal wellbeing (WHO-5: M = 70.36, SD = 15.46), self-control (M = 3.98, SD = 0.47), partner support, and friend support. In contrast, perceived stress and ADHD symptoms were generally low to moderate.

### 3.4. Preliminary Analysis

Prior to hypothesis testing, regression assumptions were evaluated. Visual inspection of histograms, Q–Q plots, detrended Q–Q plots, and standardized residual plots indicated no substantial departures from normality, linearity, or homoscedasticity. Standardized residuals ranged from −2.35 to 3.18, remaining within acceptable limits. Cook’s distance values were low (maximum = 0.165), indicating that no single observation exerted undue influence on the model, whereas the largest Mahalanobis distance (28.44) did not suggest the presence of influential multivariate outliers. Multicollinearity diagnostics were also satisfactory, with variance inflation factors (VIFs) ranging from 1.06 to 6.04. A summary of the diagnostic statistics is presented in Table 3, and graphical diagnostics are provided in Supplementary Figure S1.

To increase the robustness of parameter estimation, bias-corrected and accelerated (BCa) bootstrap confidence intervals based on 5000 resamples were calculated for all regression coefficients. The bootstrap results were consistent with conventional significance testing, supporting the stability of the reported findings.

### 3.5. Hierarchical Regression Predicting Maternal Wellbeing

A hierarchical multiple regression analysis was conducted to examine the associations between demographic characteristics, psychological vulnerabilities, self-control, perceived social support, and maternal wellbeing. Results for all regression models are presented in Table 4.

In Model 1, age and maternal education were entered as control variables. This model was not statistically significant, F(2, 146) = 0.72, *p* = 0.488, accounting for approximately 1% of the variance in maternal wellbeing (R^2^ = 0.010).

Model 2 added perceived stress and ADHD symptoms. This step substantially improved model fit, explaining an additional 35.1% of the variance in maternal wellbeing (ΔR^2^ = 0.351, Fchange = 39.51, *p* < 0.001). Perceived stress emerged as a strong negative predictor of wellbeing, whereas ADHD symptoms did not reach statistical significance after controlling for the remaining variables.

In Model 3, self-control was introduced. Although the explained variance increased slightly (R^2^ = 0.363), the addition of self-control did not significantly improve model fit (ΔR^2^ = 0.003, Fchange = 0.59, *p* = 0.445). The linear self-control term was not a significant predictor of maternal wellbeing after accounting for perceived stress and ADHD symptoms.

Model 4 introduced perceived support from partners, family members, and friends. The inclusion of these variables resulted in only a marginal increase in explained variance (R^2^ = 0.367), and the change in model fit was not statistically significant (ΔR^2^ = 0.004, Fchange = 0.28, *p* = 0.842). None of the social support variables emerged as significant predictors at this stage of the analysis.

Finally, Model 5 included quadratic terms for self-control, perceived stress, partner support, family support, and friend support to test the Too-Much-of-a-Good-Thing (TMGT) hypothesis. The inclusion of these nonlinear terms significantly improved model fit, explaining an additional 8.7% of the variance in maternal wellbeing (ΔR^2^ = 0.087, Fchange = 4.30, *p* = 0.001). The final model accounted for 45.4% of the variance in maternal wellbeing (R^2^ = 0.454, Adjusted R^2^ = 0.401).

Within the final model, perceived stress remained the strongest predictor of lower maternal wellbeing (B = −17.75, β = −0.51, *p* < 0.001, 95% CI [−23.44, −12.06]). Family support emerged as a significant positive predictor of maternal wellbeing (B = 6.73, β = 0.26, *p* = 0.039, 95% CI [0.35, 13.11]), indicating that mothers who perceived greater support from family members reported higher levels of wellbeing. ADHD symptoms approached statistical significance but did not meet conventional significance thresholds (*p* = 0.099). No significant effects were observed for age, maternal education, self-control (linear), partner support, or friend support.

**Figure 1:**
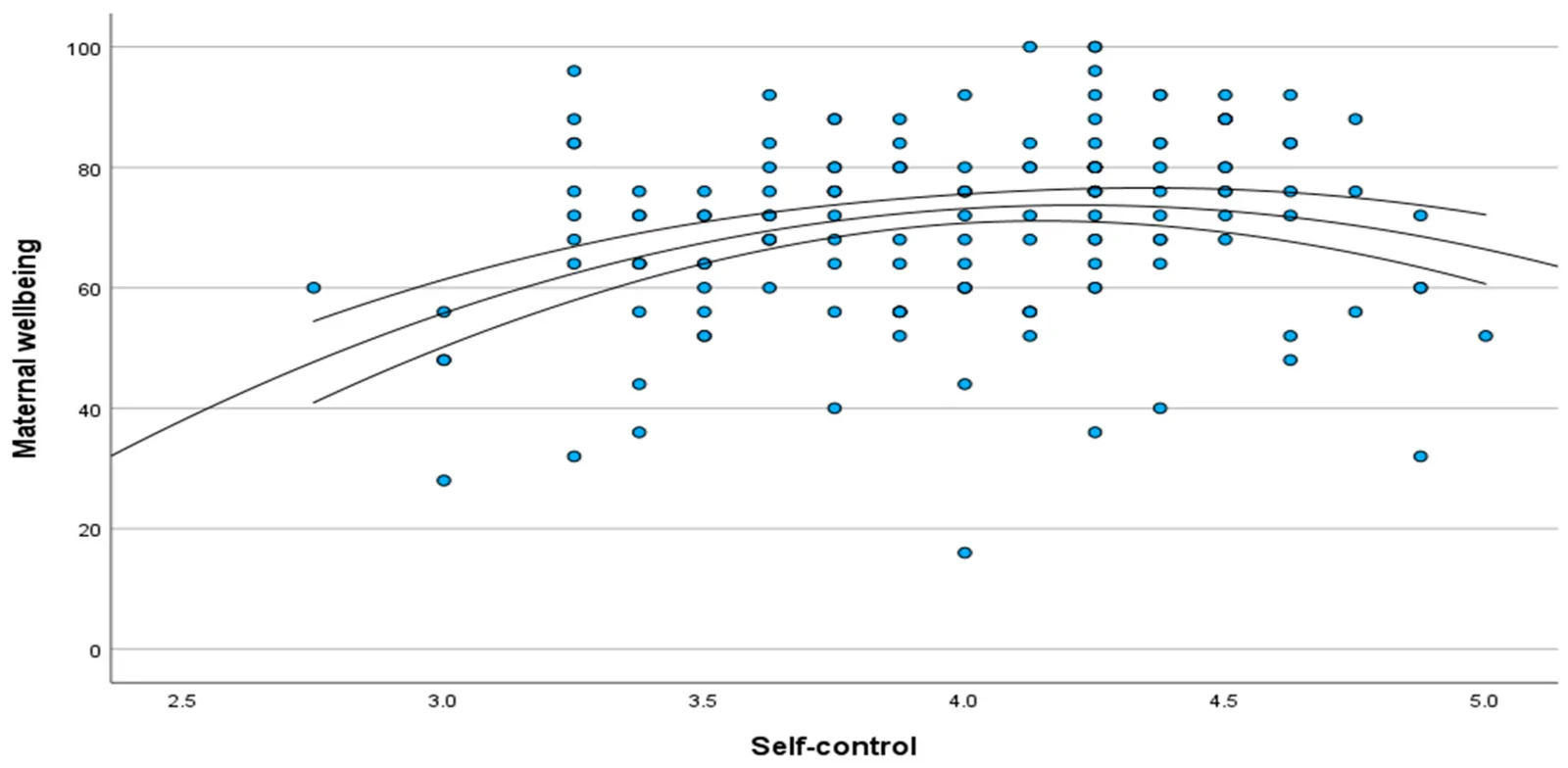
Observed association between self-control and maternal wellbeing, with an unadjusted quadratic fit and 95% confidence band for the mean fitted response. Inferential estimates are based on the adjusted hierarchical regression model reported in Table 4.

Among the nonlinear predictors, only the quadratic self-control term emerged as significant (*B* = −12.55, β = −0.21, *p* = 0.001, 95% bootstrap CI [−20.43, −4.42]). The estimated vertex of the quadratic function occurred at approximately −0.16 centered self-control units, indicating that the estimated turning point occurred at a level of self-control slightly below the sample mean. Importantly, this turning point fell within the observed range of centered self-control scores (−1.23 to 1.02). The nonlinear association between self-control and maternal wellbeing is illustrated in Figure 1. Inspection of the fitted curve suggested a relatively shallow curvilinear pattern characterized by diminishing returns, such that maternal wellbeing increased as self-control increased from low to moderate levels, whereas the slope progressively flattened at higher levels of self-control. In contrast, the quadratic terms for perceived stress, partner support, family support, and friend support were all non-significant. These results indicate that perceived stress, family support, and the quadratic self-control term were the only significant predictors in the final model.

## 4. Discussion

### 4.1. Overview of the Main Findings

This study examined the associations of perceived stress, ADHD symptoms, self-control, and perceived social support with maternal wellbeing during the second trimester of pregnancy, while also testing whether self-control and social support exhibited nonlinear associations with wellbeing in line with the Too-Much-of-a-Good-Thing (TMGT) framework. Several important findings emerged. First, perceived stress was the strongest predictor of maternal wellbeing, demonstrating a substantial negative association even after controlling demographic characteristics, ADHD symptoms, self-control, and perceived social support. Second, among the social-support dimensions examined, family support emerged as a significant positive predictor of maternal wellbeing, whereas partner support and friend support did not contribute uniquely to the final model. Third, and most importantly, a significant quadratic effect of self-control was identified. The negative quadratic coefficient indicated that the positive association between self-control and maternal wellbeing became progressively weaker at higher levels of self-control, consistent with a diminishing-returns pattern. In contrast, statistically significant quadratic effects were observed for perceived stress or any dimension of perceived social support (Arbel et al., 2022).

Although ADHD symptoms were negatively associated with maternal wellbeing at the bivariate level, they did not retain a unique association in the final multivariable model. This result should be interpreted cautiously in light of the psychometric properties of the adapted ADHD measure. Its brevity and mixed item sources may have introduced measurement error, attenuating the regression coefficient and reducing statistical power to detect a unique association after adjustment for the other predictors (Glas et al., 2024). In addition, the overlap between ADHD symptoms and perceived stress may have reduced the variance uniquely attributable to ADHD when both variables were included in the model. Accordingly, the nonsignificant multivariable coefficient should not be interpreted as definitive evidence that ADHD symptoms are unrelated to maternal wellbeing. Replication using a fully validated adult ADHD instrument or a gold-standard diagnostic assessment is needed (D. A. Diallo et al., 2004).

Overall, these findings suggest that maternal wellbeing is influenced by both psychological vulnerabilities and psychosocial resources, but that these resources may not operate according to identical mechanisms. Whereas family support exhibited a positive linear association with wellbeing, self-control demonstrated a nonlinear pattern consistent with key propositions of the TMGT framework.

### 4.2. Perceived Stress as the Strongest Predictor of Maternal Wellbeing

Perceived stress emerged as the strongest predictor of maternal wellbeing throughout all regression models. Mothers who reported higher levels of perceived stress consistently reported lower levels of psychological wellbeing, even after controlling for demographic characteristics, ADHD symptoms, self-control, and perceived social support.

This finding is consistent with a substantial body of perinatal research identifying perceived stress as a key correlate of maternal mental health during pregnancy. Elevated levels of perceived stress have been associated with lower psychological wellbeing, increased depressive and anxiety symptoms, and poorer emotional adjustment across the perinatal period (Biaggi et al., 2016; Faleschini et al., 2020; Yildiz et al., 2017). Pregnancy involves numerous physiological, psychological, and social changes, and women who perceive these demands as exceeding their available coping resources may be particularly vulnerable to emotional distress. Within the transactional model of stress, perceived stress reflects not only exposure to challenging circumstances but also individuals’ appraisal of their ability to manage those demands, making it closely linked to psychological wellbeing (Lazarus & Folkman, 1984; Giurgescu & Misra, 2018).

The present result is also consistent with emerging Romanian evidence. In a second-trimester sample of primiparous women, perceived stress and other psychological vulnerabilities were associated at the bivariate level with adverse obstetric outcomes, although generalized anxiety was the only independent psychological predictor after the psychological variables were considered simultaneously (Socol et al., 2025). By identifying perceived stress as the strongest predictor of positive psychological functioning, the present study complements this work and extends Romanian evidence beyond obstetric complications to maternal wellbeing as an outcome in its own right.

Several mechanisms may explain this association. Elevated stress has been linked to increased emotional exhaustion, reduced positive affect, impaired emotion regulation, and diminished capacity to engage in restorative activities (Park et al., 2018). Chronic stress may also interfere with mothers’ ability to perceive and utilize available social and personal resources effectively, thereby amplifying its impact on overall wellbeing.

The present findings therefore reinforce the view that stress reduction remains an important target for interventions designed to promote maternal mental health and wellbeing during pregnancy (Bonanno & Burton, 2013).

### 4.3. The Nonlinear Association Between Self-Control and Maternal Wellbeing

The most theoretically important finding of this study was the identification of a significant nonlinear association between self-control and maternal wellbeing. Consistent with previous research, higher levels of self-control were generally associated with better psychological functioning (de Ridder et al., 2012; Tangney et al., 2004). Importantly, however, the significant negative quadratic term suggests that the benefits of self-control were not uniform across its entire range but instead followed a pattern of diminishing returns, whereby higher levels of self-control were associated with progressively smaller improvements in maternal wellbeing (B. V. Radu & Haruța, 2025; Strube et al., 2017).

Several theoretical explanations may account for this pattern. Although self-control is generally associated with adaptive functioning, excessively rigid self-regulation may reduce flexibility in responding to changing situational demands. High levels of self-control may be accompanied by overregulation, perfectionistic tendencies, or excessive self-monitoring, all of which can limit psychological wellbeing when maintained inflexibly (H. T. Lynch et al., 2015; Kashdan & Rottenberg, 2010). Consistent with this perspective, psychological flexibility has been identified as a key component of mental health, enabling individuals to adapt their responses to changing environmental demands rather than relying exclusively on rigid self-control strategies (Gloster et al., 2017; Kashdan & Rottenberg, 2010).

Although this interpretation is theoretically plausible, it should also be considered in light of the psychometric properties of the self-control measure. Although McDonald’s omega suggested acceptable composite reliability, the relatively modest Cronbach’s alpha coefficient suggests that measurement error may have attenuated both the linear and nonlinear associations involving self-control. Previous research has shown that measurement error reduces regression coefficients and statistical power, thereby increasing the likelihood of underestimating true relationships in regression analyses (Glas et al., 2024). Furthermore, in quadratic regression models, measurement error may flatten the estimated regression function and reduce the apparent curvature of nonlinear effects (Kuha & Temple, 2003). Consequently, although the observed nonlinear association remained statistically significant, its precise magnitude should be interpreted with appropriate caution until replicated in independent samples.

Furthermore, although the estimated turning point of the quadratic function fell within the observed range of self-control scores, the fitted curve exhibited only a relatively shallow curvature. Accordingly, the observed pattern appears more consistent with diminishing returns or a plateau than with a pronounced inverted-U relationship. Therefore, the present findings should be interpreted as providing tentative rather than definitive support for the Too-Much-of-a-Good-Thing framework. Future research should determine whether this pattern reflects a true quadratic association or a more gradual plateau by replicating these findings in larger samples and applying alternative approaches for modelling nonlinear associations (Grote & Berens, 2024; Guo & Dipietro, 2010).

Importantly, the present findings should not be interpreted as suggesting that high levels of self-control are detrimental to maternal wellbeing. Rather, they indicate that the psychological benefits associated with self-control may eventually plateau, supporting the notion that psychological strengths may exhibit limits beyond which additional gains become increasingly modest.

### 4.4. Family Support as a Protective Resource for Maternal Wellbeing

Unlike partner support and friend support, family support emerged as a significant positive predictor of maternal wellbeing in the final model. Mothers who perceived greater support from family members reported higher levels of wellbeing, even after controlling for psychological vulnerabilities and other forms of social support.

The broader relevance of relational and contextual resources is also reflected in recent Romanian research. A large national study found that educational attainment, stable employment, and partner support were associated with more favorable quality-of-life profiles during pregnancy (C. P. Radu et al., 2025). Romanian research has also extended the study of maternal wellbeing to postpartum sexual quality of life and to the social and obstetric vulnerabilities associated with adolescent pregnancy (Brezeanu et al., 2025). These latter studies address different populations, stages of the perinatal period, and outcomes and therefore cannot be directly compared with the present sample. Nevertheless, they broaden the Romanian evidence base by illustrating that maternal wellbeing is multidimensional and is shaped by relational, sociodemographic, developmental, and obstetric circumstances across the perinatal continuum.

This finding aligns with social-support theories emphasizing the protective role of close familial relationships during periods of elevated stress and role demands (Cohen & Wills, 1985). The absence of significant effects for partner and friend support contrasts with some previous studies reporting beneficial associations between these forms of support and maternal mental health during pregnancy (Biaggi et al., 2016; Stapleton et al., 2012). However, findings across the literature have not been entirely consistent, and the relative importance of different support sources appears to vary across populations, stages of pregnancy, and outcome measures. In the present study, family support emerged as the only social-support dimension that contributed uniquely to maternal wellbeing after accounting for perceived stress, ADHD symptoms, self-control, and the remaining support variables, suggesting that family relationships may represent a particularly relevant source of support during the second trimester of pregnancy.

This pattern may also be interpreted in light of the Romanian sociocultural context. Romania has been characterized as a relatively strong-family setting, in which mutual intergenerational support and family-care obligations remain important features of family life (Castiglioni et al., 2016). Such family networks may provide pregnant women with practical assistance, emotional reassurance, and continuity of support extending beyond the romantic partnership. However, this interpretation remains tentative because the present study did not directly assess family norms, intergenerational co-residence, or the specific forms and frequency of support exchanges. Moreover, recent Romanian evidence indicates that partner support is also positively associated with quality of life during pregnancy (C. P. Radu et al., 2025). Therefore, the unique contribution of family support in the present sample should not be interpreted as evidence that partner support is generally less important in Romania; it may also reflect shared variance among support sources, restricted variability, or the particular characteristics of this highly educated and self-selected sample.

The absence of significant quadratic effects for the social-support dimensions may also reflect the content of the measures used. The support scales primarily assessed the perceived availability and adequacy of support from partners, family members, and friends, but did not directly capture potentially excessive, intrusive, unsolicited, or autonomy-limiting forms of involvement. Consequently, very high scores represented strongly positive perceptions of support rather than experiences of receiving “too much” support. This measurement approach may therefore have limited the ability to detect TMGT-type nonlinear associations for social support.

These findings therefore suggest that family support represents an important protective resource for maternal wellbeing, even when individual psychological vulnerabilities are considered.

### 4.5. Theoretical Implications

This study contributes to the growing literature on the Too-Much-of-a-Good-Thing (TMGT) framework by demonstrating that nonlinear effects may depend on the nature of the psychological resource under consideration. A significant nonlinear association was observed only for self-control, whereas family support, partner support, friend support, and perceived stress showed no evidence of comparable quadratic effects. These findings suggest that psychological resources are not governed by identical mechanisms and that the applicability of TMGT may vary across constructs. Specifically, self-control exhibited a pattern of diminishing returns, whereas social-support resources appeared to operate more linearly within the observed range. More broadly, the findings extend TMGT beyond its traditional focus on organizational contexts (Pierce & Aguinis, 2013) and highlight the importance of considering not only whether psychological resources are beneficial, but also whether their benefits continue to increase at the same rate across the entire range of the construct (Neff, 2003).

### 4.6. Practical Implications

Several practical implications emerge from the study findings. First, given that perceived stress emerged as the strongest negative predictor of maternal wellbeing, stress reduction should remain a primary focus of psychological support during pregnancy. Interventions may include cognitive-behavioral approaches, mindfulness-based stress reduction, prenatal mindfulness programs, psychoeducation, and strategies aimed at strengthening coping resources. Mindfulness- and acceptance-based approaches may be particularly relevant because they can reduce stress reactivity while also promoting psychological flexibility and a less rigid response to distressing thoughts, emotions, and bodily sensations.

Second, self-control should be encouraged but not idealized as a resource with unlimited benefits. The observed diminishing-returns pattern suggests that interventions should foster adaptive self-regulation while simultaneously promoting psychological flexibility and self-compassion. Such approaches should not aim to reduce self-control itself, but rather to help women maintain its adaptive benefits without relying on excessive self-monitoring, perfectionistic tendencies, or inflexible control strategies. Mothers may benefit not only from developing greater discipline and organization but also from learning when flexibility, acceptance, and emotional openness are more appropriate responses to changing pregnancy-related demands.

Third, the significant role of family support underscores the value of strengthening family-based support systems. Programs designed to enhance family communication, shared caregiving responsibilities, emotional reassurance, and practical assistance may contribute meaningfully to maternal wellbeing.

Overall, the findings suggest that effective interventions should combine stress reduction with the cultivation of flexible personal resources and the strengthening of supportive interpersonal environments.

### 4.7. Strengths and Limitations

This study has several strengths. It represents one of the few studies to examine the Too Much of a Good Thing (TMGT) framework in the context of maternal wellbeing during pregnancy. The use of hierarchical regression enabled the incremental evaluation of demographic characteristics, psychological vulnerabilities, personal resources, and social support variables. Furthermore, the inclusion of quadratic terms allowed for the direct examination of nonlinear associations, while bootstrap procedures enhanced the robustness of statistical inference. Finally, extensive diagnostic analyses indicated that the assumptions underlying the regression models were adequately satisfied.

Several limitations should also be acknowledged. First, the cross-sectional design precludes causal inference. Second, all measures relied on self-report methodology and may therefore be susceptible to shared-method variance and response biases. Third, ADHD symptoms were assessed using the same five-item composite previously employed in the Evidence for Better Lives Study (EBLS; Murray et al., 2022). Although this composite has been used in an international cohort of pregnant women, it has not yet been validated against a gold-standard diagnostic assessment for ADHD. Its brevity, mixed item sources, and relatively modest internal consistency may have introduced measurement error, attenuated the estimated associations involving ADHD symptoms, and partly contributed to ADHD symptoms not retaining a unique association with maternal wellbeing in the final multivariable model. Shared variance with perceived stress may also have reduced the variance uniquely attributable to ADHD symptoms. In addition, the relatively low levels of ADHD symptoms observed in this community sample may have restricted variability and further reduced the likelihood of detecting associations with maternal wellbeing. Consequently, these findings may not generalize to pregnant women with clinically diagnosed ADHD or more pronounced symptomatology. Findings involving ADHD symptoms should therefore be interpreted cautiously, and future studies should employ a fully validated adult ADHD instrument or a gold-standard diagnostic assessment and replicate the analyses in samples specifically recruited to include women with ADHD.

Fourth, although the sample size permitted estimation of the planned regression models, the sensitivity analysis indicated limited power to detect very small nonlinear effects. Because five quadratic terms were estimated simultaneously in Model 5, weaker unique associations, including a potentially small quadratic effect of perceived stress, may not have been detected. Accordingly, the nonsignificant quadratic coefficients should not be interpreted as conclusive evidence that nonlinear associations are absent. Replication in larger samples is needed to estimate smaller higher-order effects with greater precision and stability.

Fifth, participants were recruited exclusively through a Facebook support group for first-time pregnant women, resulting in a self-selected sample that may not be representative of the broader population of pregnant women. Although the sample included substantial ethnic diversity, it was relatively homogeneous with respect to high educational attainment, urban residence, perceived social support, and maternal wellbeing. These characteristics may have restricted variability in the study variables, thereby reducing the likelihood of detecting nonlinear effects and limiting the generalizability of the findings to women from rural settings, those with lower educational attainment, or populations with fewer socioeconomic and psychosocial resources.

Future research should address these limitations by employing longitudinal designs, incorporating multi-method assessments, recruiting larger and more diverse samples through a broader range of recruitment strategies, including women with clinically diagnosed ADHD or elevated ADHD symptom levels, further validating the adapted ADHD measure against established diagnostic instruments, and considering alternative statistical approaches for modelling nonlinear associations, such as spline regression or generalized additive models.

### 4.8. Directions for Future Research

Future studies should employ longitudinal designs to clarify the temporal relationships among self-control, stress, social support, and maternal wellbeing. Replication in larger and more diverse samples is also needed to determine the robustness of the observed nonlinear effect.

Additional research should investigate the mechanisms underlying the diminishing-returns pattern observed for self-control. Variables such as perfectionism, overcontrol, emotional suppression, and psychological flexibility may help explain why the benefits of self-control appear to plateau at higher levels.

Future studies should also examine whether other psychological strengths exhibit nonlinear associations with wellbeing. Such work would contribute to a more nuanced understanding of TMGT processes and help identify the conditions under which psychological resources remain beneficial versus those under which their benefits become attenuated.

## 5. Conclusions

This study examined linear and nonlinear associations between psychological vulnerabilities, psychological resources, and maternal wellbeing during the second trimester of pregnancy. Perceived stress emerged as the strongest predictor of lower maternal wellbeing, whereas family support was the only social-support dimension that contributed uniquely to wellbeing after accounting for the remaining study variables. In addition, self-control was the only variable to exhibit a significant nonlinear association with wellbeing, characterized by a pattern of diminishing returns at higher levels.

Overall, the findings suggest that maternal wellbeing is shaped by both psychological vulnerabilities and psychological resources, but that these factors may not operate through identical mechanisms. By identifying a nonlinear association for self-control but not for perceived stress or social support, the study highlights the importance of considering potential limits to otherwise beneficial psychological characteristics and extends the application of the Too-Much-of-a-Good-Thing framework to maternal wellbeing.

## Figures and Tables

**Table 1:** Internal Consistency Estimates for Study Measures.

Measure	α	ω
WHO-5 Wellbeing	0.84	0.84
Perceived Stress (PSS-10)	0.83	0.87
ADHD Symptoms	0.56	0.74
Self-Control	0.61	0.75
Partner Support	0.75	0.84
Family Support	0.90	0.94
Friend Support	0.93	0.95

*Note.* α = Cronbach’s alpha; ω = McDonald’s omega.

**Table 2:** Means, standard deviations, and Pearson correlations among study variables.

Variable	M	SD	1	2	3	4	5	6	7	8	9
1. Age	29.99	4.63	—								
2. Maternal education	12.84	1.98	−0.048	—							
3. Partner support	4.58	0.44	−0.137	−0.121	—						
4. Friend support	4.35	0.66	0.005	−0.042	0.319 **	—					
5. Wellbeing (WHO-5)	70.36	15.46	0.003	−0.099	0.166 *	0.163 *	—				
6. Perceived stress	1.64	0.44	−0.039	0.038	−0.275 **	−0.215 **	−0.585 **	—			
7. Self-control	3.98	0.47	0.093	0.086	0.221 **	0.186 *	0.209 *	−0.377 **	—		
8. ADHD symptoms	2.03	0.57	−0.026	−0.064	−0.179 *	−0.082	−0.290 **	0.328 **	−0.484 **	—	
9. Family support	4.63	0.60	−0.015	−0.038	0.302 **	0.586 **	0.122	−0.118	0.164 *	−0.077	—

Note. *N* = 149. WHO-5 = World Health Organization-Five Well-Being Index. * *p* < 0.05, ** *p* < 0.01.

**Table 3:** Regression Diagnostic Statistics.

Diagnostic Statistic	Value
Standardized residuals (range)	−2.35 to 3.18
Maximum Cook’s distance	0.17
Maximum Mahalanobis distance	28.44
Variance inflation factor (VIF) range	1.06–6.04

*Note.* Standardized residuals fell within acceptable limits (±3.29). Cook’s distance values were well below the recommended threshold of 1.00, indicating no influential observations. Variance inflation factors (VIFs) indicated no evidence of problematic multicollinearity (Field, 2018; Hair et al., 2019).

**Table 4:** Hierarchical regression predicting maternal wellbeing (WHO-5).

Predictor	Model 1	Model 2	Model 3	Model 4	Model 5
Age	−0.002	−0.025	−0.020	−0.024	0.027
Maternal Education	−0.099	−0.087	−0.083	−0.084	−0.118
Perceived Stress	—	−0.544 ***	−0.559 ***	−0.559 ***	−0.509 ***
ADHD Symptoms	—	−0.118	−0.142	−0.145	−0.128
Self-Control	—	—	−0.061	−0.067	−0.120
Partner Support	—	—	—	−0.034	0.079
Family Support	—	—	—	0.049	0.261 *
Friend Support	—	—	—	0.022	0.055
Self-Control^2^	—	—	—	—	−0.213 **
Perceived Stress^2^	—	—	—	—	−0.136
Partner Support^2^	—	—	—	—	0.127
Family Support^2^	—	—	—	—	0.172
Friend Support^2^	—	—	—	—	0.114
*R* ^2^	0.010	0.361	0.363	0.367	0.454
Adjusted *R*^2^	−0.004	0.343	0.341	0.331	0.401
Δ*R*^2^	0.010	0.351	0.003	0.004	0.087
*F* Change	0.721	39.505	0.587	0.277	4.302
*p* for Δ*F*	0.488	<0.001	0.445	0.842	0.001

**Note.** Values are standardized regression coefficients (β). *N* = 149. WHO-5 = World Health Organization-Five Well-Being Index; ADHD = attention-deficit/hyperactivity disorder. Model 1 included age and maternal education. Model 2 added perceived stress and ADHD symptoms. Model 3 added self-control. Model 4 added partner, family, and friend support. Model 5 added quadratic terms for self-control, perceived stress, partner support, family support, and friend support. * *p* < 0.05, ** *p* < 0.01, *** *p* < 0.001.

## Data Availability

The data that support the findings of this study are available from the corresponding author upon reasonable request.

## Supplementary Materials

The following supporting information can be downloaded at: https://www.mdpi.com/article/10.3390/bs16081380/s1, Figure S1: Diagnostic plots for the final hierarchical regression model. (A) Histogram of standardized residuals, (B) normal P-P plot of standardized residuals, (C) detrended normal P-P plot of standardized residuals, and (D) standardized residuals plotted against standardized predicted values. Visual inspection indicated no substantial departures from normality, linearity, or homoscedasticity.
