# Supplementary material for: When More Is Not Always Better: Linear and Nonlinear Predictors of Maternal Wellbeing During Pregnancy
Source: Behav Sci (Basel). 2026 Aug 11;16(8):1380. doi: 10.3390/bs16081380 (PMC13509218; doi:10.3390/bs16081380)
Supplement: Supplementary file 1 [file behavsci-16-01380-s001.zip › behavsci-4412016-supplementary.pdf]

Fig.A

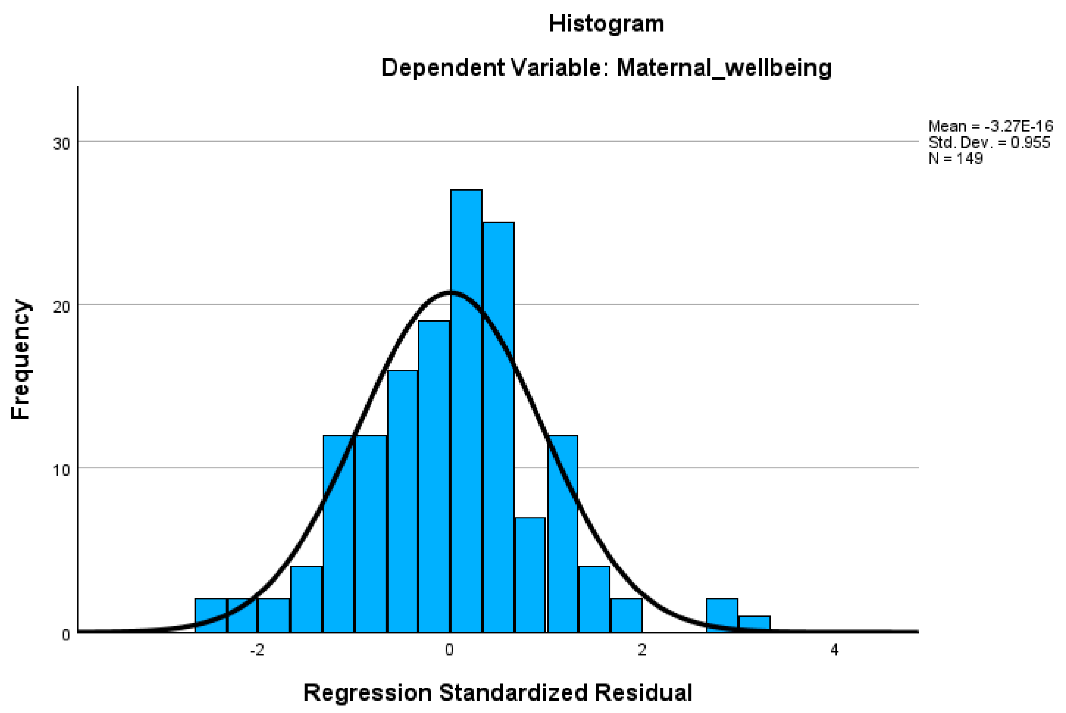
Histogram
Dependent Variable: Maternal_wellbeing
Mean = -3.27E-16
Std. Dev. = 0.955
N = 149
Frequency
30
20
10
0
-2
0
2
4
Regression Standardized Residual

Fig.B

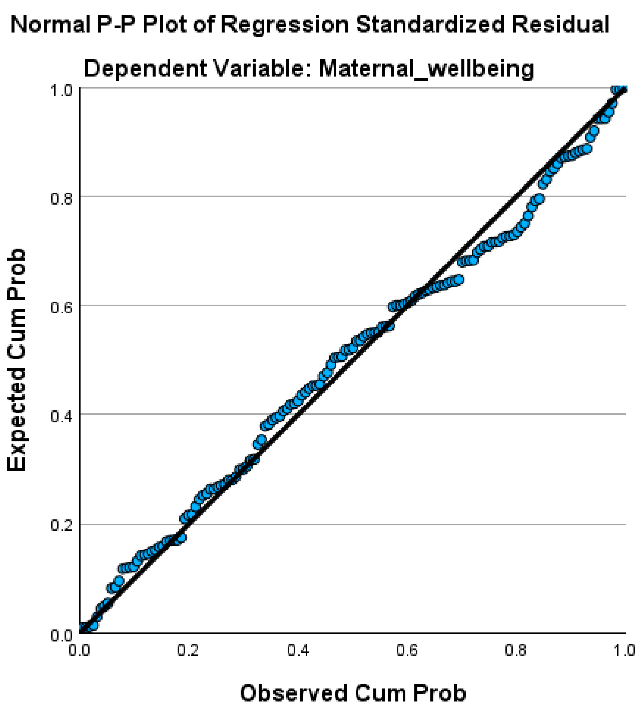
Normal P-P Plot of Regression Standardized Residual
Dependent Variable: Maternal_wellbeing
Expected Cum Prob
Observed Cum Prob
0.0
0.2
0.4
0.6
0.8
1.0

Fig.C

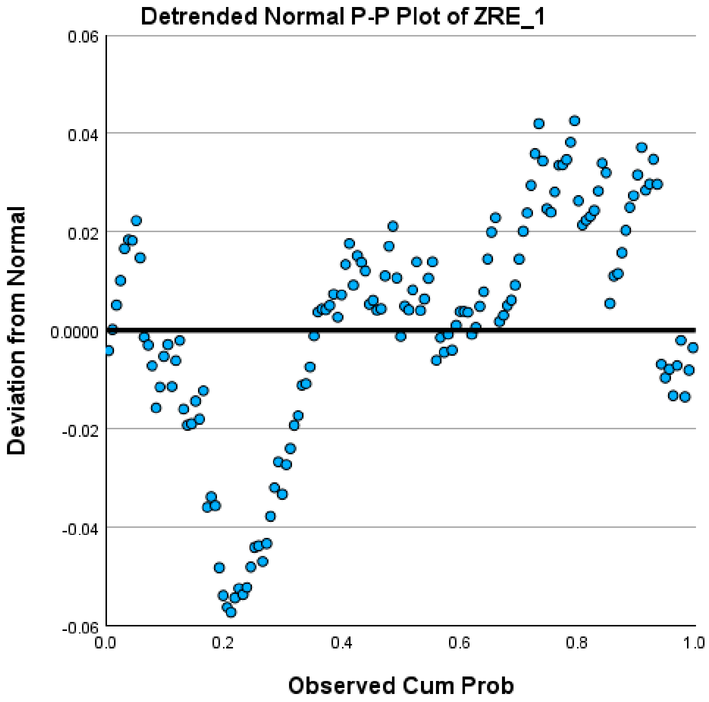

Detrended Normal P-P Plot of ZRE_1
0.06
0.04
0.02
0.0000
-0.02
-0.04
-0.06
0.0
0.2
0.4
0.6
0.8
1.0
Deviation from Normal
Observed Cum Prob

Fig.D

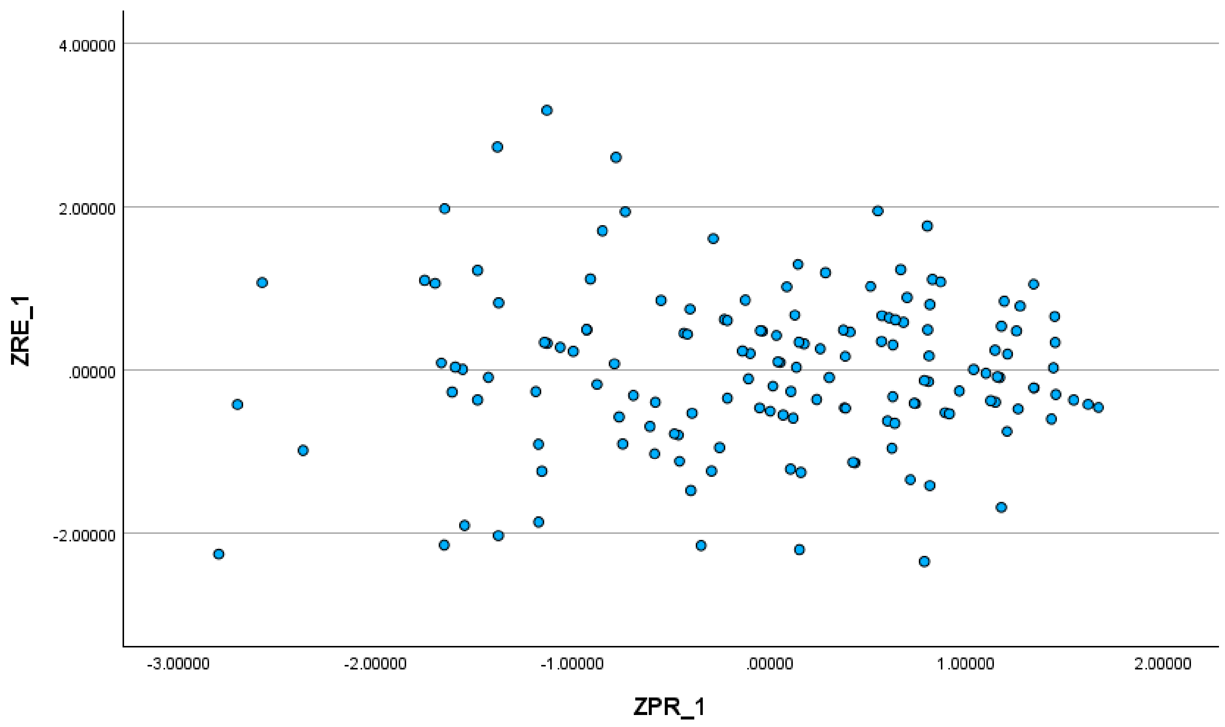

Figure S1. Diagnostic plots for the final hierarchical regression model. (A) Histogram of standardized residuals, (B) normal P-P plot of standardized residuals, (C) detrended normal P-P plot of standardized residuals, and (D) standardized residuals plotted against standardized predicted values. Visual inspection indicated no substantial departures from normality, linearity, or homoscedasticity.
